# Phospholipid production and signaling by a plant defense inducer against *Podosphaera xanthii* is genotype-dependent

**DOI:** 10.1093/hr/uhae190

**Published:** 2024-07-12

**Authors:** Theoni Margaritopoulou, Eirini Baira, Christos Anagnostopoulos, Katerina-Eleni Vichou, Emilia Markellou

**Affiliations:** Laboratory of Mycology, Scientific Directorate of Phytopathology, Benaki Phytopathological Institute, Kifissia 14561, Greece; Laboratory of Toxicological Control of Pesticides, Scientific Directorate of Pesticides' Control & Phytopharmacy, Benaki Phytopathological Institute, Kifissia 14561, Greece; Laboratory of Pesticide Residues, Scientific Directorate of Pesticides' Control & Phytopharmacy, Benaki Phytopathological Institute, Kifissia 14561, Greece; Laboratory of Mycology, Scientific Directorate of Phytopathology, Benaki Phytopathological Institute, Kifissia 14561, Greece; Laboratory of Mycology, Scientific Directorate of Phytopathology, Benaki Phytopathological Institute, Kifissia 14561, Greece

## Abstract

Biotrophic phytopathogenic fungi such as *Podosphaera xanthii* have evolved sophisticated mechanisms to adapt to various environments causing powdery mildews leading to substantial yield losses. Today, due to known adverse effects of pesticides, development of alternative control means is crucial and can be achieved by combining plant protection products with resistant genotypes. Using plant defense inducers, natural molecules that stimulate plant immune system mimicking pathogen attack is sustainable, but information about their mode of action in different hosts or host genotypes is extremely limited. *Reynoutria sachalinensis* extract, a known plant defense inducer, especially through the Salicylic acid pathway in Cucurbitaceae crops against *P. xanthii*, was employed to analyze the signaling cascade of defense activation. Here, we demonstrate that *R. sachalinensis* extract enhances phospholipid production and signaling in a Susceptible to *P. xanthii* courgette genotype, while limited response is observed in an Intermediate Resistance genotype due to genetic resistance. Functional enrichment and cluster analysis of the upregulated expressed genes revealed that inducer application promoted mainly lipid- and membrane-related pathways in the Susceptible genotype. On the contrary, the Intermediate Resistance genotype exhibited elevated broad spectrum defense pathways at control conditions, while inducer application did not promote any significant changes. This outcome was obvious and at the metabolite level. Main factor distinguishing the Intermediate Resistance form the Susceptible genotype was the epigenetic regulated increased expression of a G3P acyltransferase catalyzing phospholipid production. Our study provides evidence on phospholipid-based signaling after plant defense inducer treatment, and the selective role of plant’s genetic background.

## Introduction

The domestication and continuous breeding of plant varieties for increased production and crop quality has led to a decreased plant disease resistance, compared to wild species. Numerous plant diseases, caused by a variety of pathogens, challenge crops development, and productivity. Over time, plant diseases have caused serious nutritional and economic crises, and they continue to cause significant crop losses globally until today [1]. The process of producing crops involves the intensive usage of many pesticides to guarantee an acceptable yield and quality of the end product. However, pesticides have numerous negative consequences on ecosystems, cultivated plants, and even farmers’ and consumers’ health [2]. Fungicides, a major group of pesticides with extensive use worldwide, not only integrate all the harmful effects of pesticides but also have led to the appearance of resistant and invasive fungal pathogen strains [3, 4]. The consequences of this conventional approach of crop production have made apparent the need for alternative and sustainable disease management approaches. The use of resistant hybrids, biological management of diseases, organic and integrated agricultural methods, and various combinations of these are examples of alternative methods. A promising strategy, which is compatible with organic and IPM farming and is getting continuous attention, is the use of plant protection products that function as plant defense inducers (PDIs) [1, 5]. When fungicide-based control needs to be limited, stimulating the plant immune system with these PDIs—natural molecules mimicking pathogen attack—may offer an intriguing crop protection strategy.

Most of the microbial invaders that are ubiquitously present in the environment can be encountered by the multifaceted immune system of plants. In general, induction of plant defense responses takes place upon recognition of adapted and non-adapted pathogens by a two-level innate immune system [6]. The first level of defense is referred to as PAMP/MAMP-triggered immunity (PTI/MTI) because it is triggered by plant cell surface-localized pattern recognition receptors (PRRs) recognizing conserved molecules of the pathogens, also known as pathogen- or microbial-associated molecular patterns (PAMPs or MAMPs). Secondly, plants employ cell-surface receptor-like proteins or intracellular nucleotide binding site leucine-rich-repeat (NB-LRR) proteins to identify pathogen-secreted molecules, named effectors, to suppress pathogen-induced immunity (PTI) and promote pathogenesis. This process results in the induction of defense responses known as effector-triggered immunity [6]. A variety of early signaling events including rapid accumulation of reactive oxygen species (ROS), activation of protein kinase cascades, alterations in gene expressions, and synthesis of defense-related hormones are involved in the intricate process of activating plant immunity [7, 8].

The interplay of plants with microbes is highly dynamic and can be reshaped and adapt to fluctuating environments. The plasma membrane (PM) of the plant cells, receives changes of the extracellular environment and regulates response mechanisms with a sophisticated pathway that organizes signal transduction. PM bridges microbe recognition with plant cellular responses, either allowing a synergistic interaction between the microbe and the plant host or inhibiting it by activating downstream signaling cascades [9]. Cell wall is the primary defensive barrier of the plant against environmental perturbations and provides natural defense upon microbe penetration [10]. In addition, PM plays the critical role of transmitting signaling responses due to external stimuli and microbe recognition, since microbes attempt to manipulate these pathways in order to suppress plant defense responses for successful colonization and nutrient acquisition [9, 11, 12]. Lipids are major components of the PM that provide physical barriers in the plant cell outer layers and influence the communication between the host and the microbe. Lipids also act as signaling molecules and participate in the recognition of PDIs, taking active part in the establishment or prevention of microbial colonization [13]. Nowadays, there is increasing attention in investigating how lipids and lipid-related compounds, such as phospholipids, fatty acids, sterols, and jasmonates, participate in the control of plant defense responses [14, 15]. Phospholipids, a major class of PM components, function as structural components, as signaling molecules and regulate how extracellular signals are perceived during establishing plant defense. The evidence of the dynamics of activation and translocation of phospholipids linked to plant signaling, and particularly to the recruitment of defense regulators during pathogen attack, is accumulating [16–18]. Therefore, is crucial to clarify the regulatory role of phospholipid-based signaling in in plant immunity.

In this work, we present a combination of nontargeted and targeted approaches that was used to elucidate the molecular mechanisms behind *Reynoutria sachalinensis* (Rs) treatment in courgette (*Cucurbita pepo* ssp *pepo*), an important and popular crop [19]. Leaves of Susceptible and Intermediate Resistance courgette genotypes were sprayed with Rs, as this strategy is a common practical approach in agriculture. Global transcriptomic and metabolomic analyses together with gene expression and protein quantification in courgette plants contributed to the identification of molecular mechanisms and signaling cascades that take place after Rs treatment and *Podosphaera xanthii* (Px) inoculation and how different genetic backgrounds influence these functions.

## Results

### Transcriptome modifications after PDI treatment

**Figure 1:**
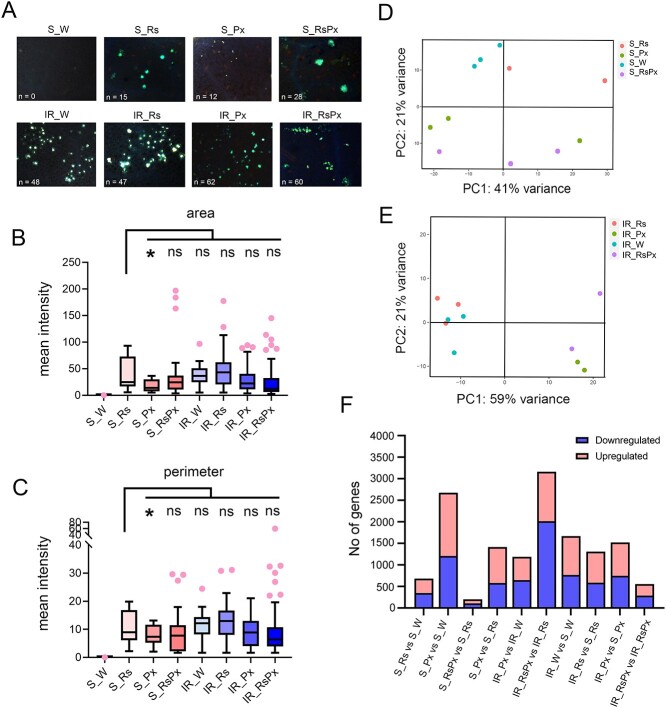
Rs extract stimulates transcriptomic changes in Susceptible to *Podosphaera xanthii* courgette genotypes. (A) Representative microscopy images (20 × magnification) of the S and IR genotype leaves where fluorescence signals detect callose deposits by Lactophenol blue staining and fluorescence microscopy. (B, C) Mean fluorescence intensity determined by ImageJ software of callose deposits area (B) and perimeter (C). Asterisks indicate statistical significance of the mean (±SE) determined in independent groups T-test, *p*-value <0.05. (D, E) PCA showing the transcriptomic effect of Rs and Px inoculation on the leaves of a Susceptible (D) and an Intermediate Defense (CE) courgette genotype. (F) Numbers of genes that are differentially expressed in each statistically valid group comparison. Plant treatments were performed with water (W), Rs, pathogen inoculation (Px), and combination of Rs and Px.

Prior knowledge has demonstrated that plant’s genetic background influences physiological changes after Rs treatment [20, 21]. To examine this, we observed callose deposition on a Susceptible (S, Kompo) and an Intermediate Resistance (IR, Cordelia) courgette genotype treated with either Rs extract (Rs) or Water (W, Control) and inoculated with Px and combination of Rs and Px. Fluorescent microscopy was applied for histochemical analysis of callose deposition and showed induced production of this compound after Rs treatment, Px inoculation or both in the S genotype as expected (Fig. 1A). On the contrary, the IR genotype displayed high callose deposition in the control plants, approximately 48 callose deposits per 0.025 mm^2^, which remained relatively constant between treatments (Fig. 1A). This callose deposition was almost 3-fold higher compared to that observed in the S genotype, while Px inoculation and Rs plus Px treatments led to a modest reduction in the size of the deposits but not their number (Fig. 1A). Area and perimeter measurements of callose deposits using ImageJ software verified the fluorescent observations (Fig. 1B and C). To investigate transcriptomic changes that Rs treatment caused in the two genotypes, we performed RNAseq. Rs was sprayed on leaves on a weekly basis and after the third treatment, artificial inoculation with Px conidia was performed. Three leaf biological replicates were sampled 36 h post inoculation (hpi) and 24 samples were collected for RNA sequencing. RNA sequencing generated high-quality reads averaging 45.142.754 reads per sample. Mapping of the reads for each sample was performed with the *C. pepo* (MU-CU-16) v4.1 reference genome, with a successful alignment of 85% of the reads to an average of 19.888.441 counts per sample (Supplementary Data Set S1). Principal Component Analysis (PCA) results revealed, in the S genotype, that Rs treatment and Px inoculation differentiated the transcriptome (Fig. 1D), while in the IR genotype, significant differentiation in expression was apparent only in Px-inoculated versus non inoculated plants with or without Rs treatment (Fig. 1E). In the subsequent comparisons we used cut-offs of Padj <0.05, and ≥ 1-fold in expression change. By these criteria, a significant number of differentially expressed genes (DEGs) was identified in comparisons where pathogen inoculation was applied, while Rs treatment did not induce high differentiation in expression (Fig. 1F, Supplementary Fig. S1 and Set S2), indicating a modest modification in expression with apparently fewer genes to be influenced.

### PDI treatment triggers the enrichment of lipid pathway in the susceptible genotype

**Figure 2:**
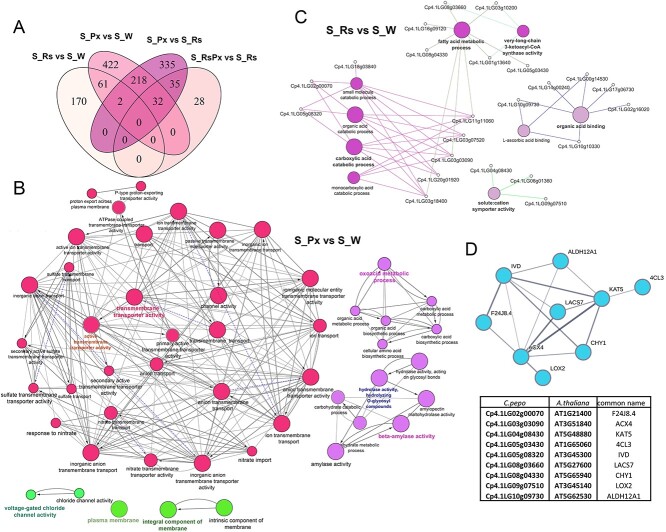
Rs extract modulates the enrichment of lipid pathways in Susceptible to Px courgette genotype. (A) Overlap between genes induced in *R. sachalinensis* treated, pathogen inoculated or combination of both Susceptible plant leaves. (B) Enriched GO terms of the pathogen inoculated versus water comparison in the Susceptible genotype and (C) of *R. sachalinensis* treated versus water comparison applying cut-offs of a ≥ 2-fold difference and *p*adj ≤ 0.05 in expression. Node size shows GO-term significance (*p-value*): smaller *p-value* is positively correlated to larger node size. Common genes are represented with lines between nodes, and thicker lines represent larger overlap. Different node groups show GO-terms classification into functional groups. Word highlight indicates the names of the most significant GO-terms for each group. Interaction networks were constructed and visualized by Cytoscape [22]. (D) Interaction network of *A. thaliana* homologous proteins to *C.pepo* proteins of the *R. sachalinensis* treated versus water differential expression results. STRING was used to construct and visualize the interaction network by a minimum required interaction score of 0.7 [23].

Our results show that Rs treatment caused limited modification on cellular processes in the S plants. To determine the differences between upregulated genes after Rs treatment and pathogen inoculation, the upregulated gene sets of the statistically valid comparisons in the S genotype was compared. We found that only 8.5% of the upregulated genes after pathogen inoculation overlapped with genes after Rs treatment, corresponding to 23.6% of this gene set (Fig. 2A). To generate the Gene Ontology (GO) enrichment network based on RNA-Seq data, we directly mapped the upregulated genes to pathways in the Cytoscape database. The plethora of the enriched GO terms associated with upregulated genes after pathogen inoculation, were strongly related to the membrane and transmembrane activities, especially to ion transports and hydrolyzing procedures (Fig. 2B, Supplementary Table S1), indicating primary defense responses to fungus infection, 36 h after inoculation. On the other hand, 23 (10%) out of the 235 upregulated DEGs after Rs treatment were significantly associated to GO terms related to fatty acid metabolism, carboxylic acid metabolism, organic acid binding, 3-ketoacyl-CoA synthase activity, and symporter activity (Fig. 2C, Supplementary Table S2). All these GO terms interfere with lipid metabolism and transmembrane compound transfer [24, 25], suggesting that the weekly applications of Rs established a distinct enrichment in plant cells. Indeed, when we directly mapped the *Arabidopsis thaliana* homolog proteins of the upregulated genes in the String database, the generated protein–protein interaction network showed significantly high network association with protein–protein interaction enrichment *p-*value = 1.07e^−14^ of nine proteins (39%), that were integrated with all the major enriched GO Terms (Fig. 2C, D).

### The IR genotype is described by highly activated defense signaling machinery particularly without Rs treatment

**Figure 3:**
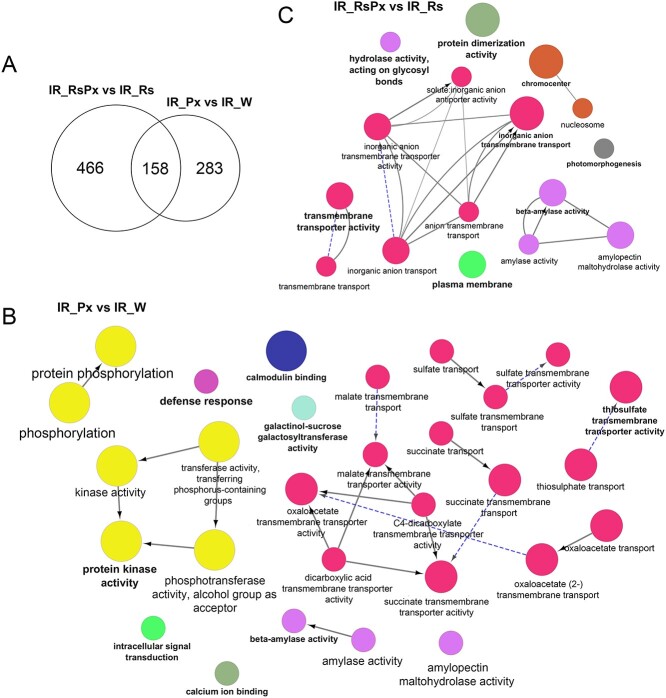
Pathogen inoculation activates inherent defense mechanisms in Intermediate defense genotype after pathogen inoculation. (A) Overlap between genes induced by pathogen inoculation in *R. sachalinensis* treated or water treated Intermediate defense plant leaves. Enriched GO terms of the pathogen inoculated versus water comparison (B) and the pathogen inoculated versus control in *R. sachalinensis* treated plant leaves (C) in the Intermediate defense genotype applying cut-offs of a ≥ 2-fold difference and Padj≤0.05 in expression. Interaction networks were constructed and visualized by Cytoscape [22]. Node size shows GO-term significance (*p-value*): smaller *p-value* is positively correlated to larger node size. Common genes are represented with lines between nodes, and thicker lines represent larger overlap. Different node colors show GO-terms classification into functional groups. Word highlight indicates the names of the most significant GO-terms for each group.

Gene sets with specifically increased transcript abundance at 36 hpi were examined for overlapping in the IR genotype with and without Rs treatment. Interestingly, overlapping upregulated genes between water and Rs treatment was less than 50%, while pathogen inoculation in Rs-treated leaves caused greater differential gene expression than the water-treated ones (Fig. 3A). To identify critical processes upregulated by pathogen inoculation in the IR genotype, a GO term enrichment analysis was performed. After pathogen inoculation in the water-treated samples, significant enrichment was observed in defense-related pathways such as protein kinase activity, phosphorylation, signal transduction, ion binding, and defense response, demonstrating immediate response to pathogen infection (Fig. 3B, Supplementary Table S3). Evidence on the genetic background of the IR plants have shown the heterozygous introgression of the major *Pm-0* defense locus that is epigenetically controlled and confers partial defense against Px infection [20, 26]. Surprisingly, even though gene upregulation after pathogen inoculation in Rs-treated IR plants was greater than water-treated ones (486 to 283 DEGs, respectively), the enriched GO terms were scaled down and connected to less important defense-associated networks, such as transmembrane transporter activity, hydrolase activity and PM, indicating that the combination of Rs with Px impaired defense response (Fig. 3C, Supplementary Table S4). This result is consistent with previous knowledge showing significant reduction of physiological defense-related processes after Rs treatment in IR courgette genotypes [21].

### Rs mimics pathogen infection to generate defense response

**Figure 4:**
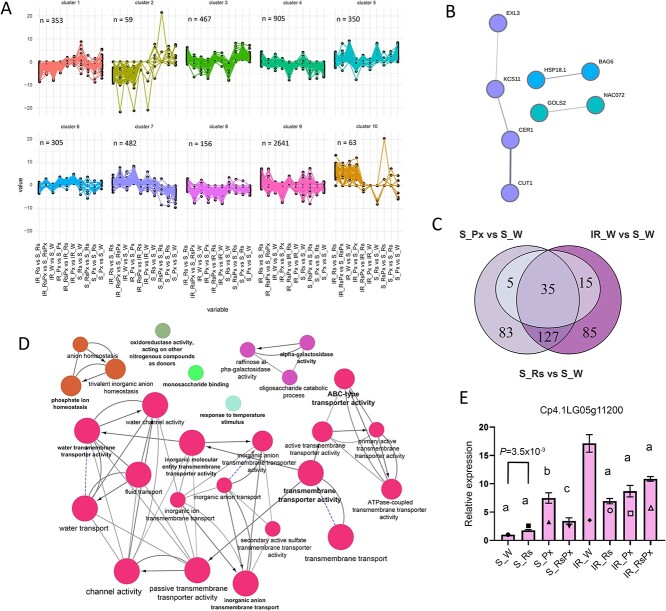
Rs PDI regulates the enrichment of lipid pathways in Susceptible to *Podosphaera xanthii* genotype. (A) K-means clustering to assess similar expression profiles in the transcriptomic results illustrating ten clusters. Each subgroup corresponds to one gene cluster showing similar profile in expression. The horizontal axes correspond to the valid expression comparisons of each genotype and between genotypes. The vertical axes depict the corresponding logarithmic ratios, as derived from the transcriptomic data analysis. n = number of genes in each cluster. (B) STRING network of the interacting homologues to *A. thaliana* proteins of the cluster 1 subgroup. (C) Overlap between genes in cluster 5 that are highly upregulated in pathogen inoculated versus water comparison and *R. sachalinensis* versus water treatment in the Susceptible genotype plant leaves, and in Intermediate defense versus Susceptible water treated plant leaves. (D) Enriched GO terms of the cluster 5 subgroup. Interaction networks were constructed and visualized by Cytoscape [22]. Node size shows GO-term significance (*p-value*): smaller *p-value* is positively correlated to larger node size. Common genes are represented with lines between nodes, and thicker lines represent larger overlap. Different node groups show GO-terms classification into functional groups. Word highlight indicates the names of the most significant GO-terms for each group. (E) Relative expression *PATATIN* (Cp4.1LG05g11200) in leaves of Susceptible and Intermediate defense plants after *R. sachalinensis* treatment, pathogen inoculation and combination of both. Data represents the mean ± SEM of six biological replicates obtained from two independent experiments. *p-value*s are determined by Brown–Forsythe ANOVA with a post hoc Dunnett’s multiple comparison test. Different letters indicate significant differences. Difference between S_Rs and S_W was determined by two-tailed Student’s t-test with Welch correction. Geometrical symbols on each sample bar represent relative to S_W normalized counts.

To understand in depth the Rs mode of action and how its correlated with pathogen infection and genetic background, we searched for candidate targets of putative, common regulatory mechanisms by assessing similar expression profiles in the RNAseq results through Kmeans clustering. We performed a thorough examination of DEGs in all statistically valid expression comparisons of each genotype and between genotypes. Based on Within Sum of Squares plot, DEGs were divided into 10 clusters (Fig. 4A, Supplementary Data Set S3). We focused on subgroups of coregulated DEGs where expression fold change had a positive increment mainly in the comparisons of Rs versus water and Px versus water in the S genotype, and IR-treated versus S-treated genotypes. Analysis of cluster 1 coregulated DEGs revealed substantial upregulation mainly in Rs versus water comparison of the S genotype that were highly associated with defense responses (Fig. 4A, Supplementary Table S5). Even though functional enrichment analysis of the upregulated genes in this comparison, did not show any significant enrichment, 9 (20%) out of 47 genes were related to fatty acid signaling (Supplementary Table S5). STRING analysis of *A. thaliana* homologues proteins showed that 3-KETOACYL-COA SYNTHASE 11 (KCS11), 3-KETOACYL-COA SYNTHASE 6 (CUT1), GDSL ESTERASE/ ACYLTRANSFERASE/ LIPASE (EXL3), and ECERIFERUM 1 (CER1) interact physically and are components of the fatty acid pathway (Fig. 4B, Supplementary Table S5), indicating a substantial link between Rs treatment and induction of fatty acids. Two other groups of the examined proteins were the small heat shock protein HSP18.1 that interacted with BCL-2-ASSOCIATED ATHANOGENE 6 (BAG6) that regulate stress responses [27], and GALACTINOL SYNTHASE (GLOS2) with NAC DOMAIN-CONTAINING PROTEIN 72 (NAC72) that are components of oligosaccharide biosynthesis.

The finding of the coregulated genes in cluster 5 were of particular interest. In this subgroup a significant fraction of 162 genes (46%) was common with high expression upregulation in Px versus the S genotype water-treated plants and IR-treated versus S-treated genotype comparisons, and even though Rs versus water in S genotype comparison revealed 55 genes, 64% of them overlapped within the other comparisons (Fig. 4C). Examination of the functional enrichment of the genes in this cluster revealed the essential role of transmembrane transporter activity since the plethora of enriched terms, 18 out of 27 terms, were integrated with this function (Fig. 4D, Supplementary Table S6). Interestingly, this cluster included *PATATIN* (Cp4.1LG05g11200) gene, homolog to *PHOSPHOLIPASE A 2A* (*PLA2A*, At2g26560) in *A. thaliana*, with similar expression pattern in all three substantial comparisons. *PATATIN* belongs to phospholipase A2 (PLA2) superfamily with wide substrate specificity catalyzing the cleavage of diacyl-phospholipid producing lysophospholipid (LPL) and free fatty acid. *PLA2A* expression is accumulated as response to a variety of cellular processes, such as growth, development, stress responses, and defense, against fungal and bacterial pathogens [28–30]. There is accumulating evidence indicating that PLA along with integrated compounds, such as free fatty acids, LPLs, and the phospholipase C, play an important role in controlling defense response of plants against pathogen invasion [18], and our results indicate that the Rs PDI can also activate this network in plants. To validate *PLA2A* expression pattern among comparisons, we performed qRT-PCR that gave similar results with the RNAseq data (Fig. 4E).

The genetic diversity of the IR genotype was depicted in cluster 10, where the genes demonstrated high transcript abundance in the IR genotype in all treatments compared to the S genotype (Fig. 4A, Supplementary Data Set S3). Among the 63 genes in this cluster, we identified *PATHOGENESIS RELATED 4*, *THAUMATIN*, *SUPEROXIDE DISMUTASE*, *PEROXIDASE* among others that are strongly associated with pathogen resistance.

### Rs stimulates phospholipid production in the S genotype

**Figure 5:**
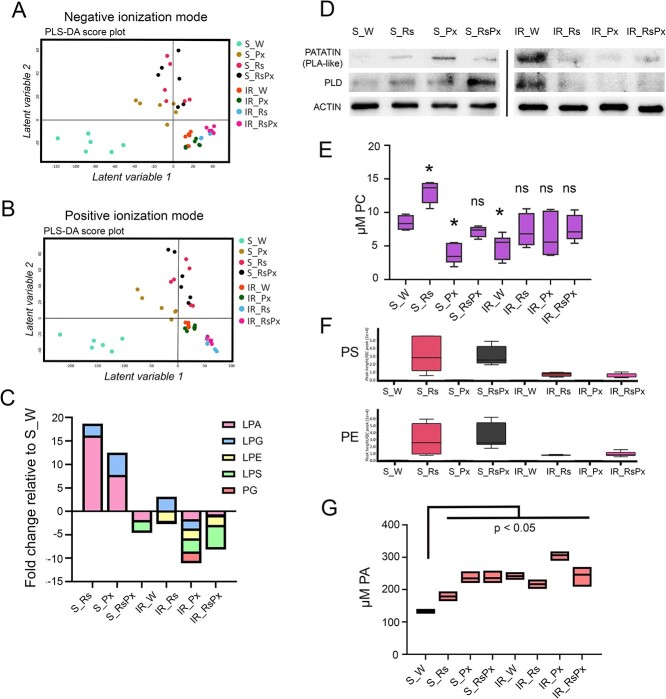
Rs extract increases glycerophospholipid production in a Susceptible to *Podosphaera xanthii* genotype. Partial least squares discriminant analysis (PLS-DA) score plot showing the metabolomic differences of the detected metabolites in the leaves of the Susceptible and Intermediate Defense genotype after Rs treatment, pathogen inoculation and combination of both in negative (A) and positive (B) ionization mode. (C) Glycerophospholipid classes that are detected and annotated in the negative ionization mode. The y axis refers to relative glycerophospholipid accumulation fold change in the statistically significant different treatments compared to S_W where LPA: Lyso-Phosphatidic acid, LPG: Lyso-Phosphatidylglycerol, LPE: Lyso-Phosphatidlyethanolamine, LPS: Lyso-Phosphatidylserine, PG: Phosphatidylglycerol. (D) Western blot of PATATIN and PLD proteins in leaves of the S and IR genotypes after the above-mentioned treatments; endogenous levels of ACTIN protein are used as internal normalization control. (E) Phosphatidylserine class that was detected in leaves of the above-mentioned treatments. Significant differences are indicated with asterisk (*p-value* < 0.05): ns denotes no statistical differences. (F) Glycerophospholipid classes that were detected in leaves of the above-mentioned treatments and annotated in the positive ionization mode where PS: Phosphatidylserine, PE: Phosphatidylethanolamine. (G) Phosphatidic acid that was quantified in leaves of the above-mentioned treatments.

Our transcriptomic findings revealed clear stimulation of the membrane-lipid machinery after Rs treatment in the S genotype. To correlate gene expressions with the biochemical landscape, we performed whole untargeted metabolomic analysis to search for lipid compound variations. In the negative ionization mode, only Rs treatment resulted in significant separation of the metabolic profiles in both tested genotypes, while in positive ionization mode such separation was evident only in S genotype (Fig. 5A and B). The results were matched to Lipid Maps database and at the negative mode, Lyso-Phospatidic acid (LPA), and Lysophosphatidylglycerol (LPG) classes were annotated with 16- and 2.5-fold change in S_Rs and 7.5- and 4-fold change increase in S_RsPx samples, respectively (Fig. 5C, Supplementary Data Set S4). It is known that LPA is *de novo* produced from glycerol-3-phosphate (G3P) by G3P acyltransferase to be converted to Phosphatidic acid (PA) [31, 32], or from PA by PLA to serve as lipid storage [17, 33]. Thus, LPA accumulation in correlation with *PLA* upregulated gene expression in S_Rs genotype reinforces the interplay of Rs treatment with lipid signaling. To verify this, we analyzed PATATIN at the protein level. We detected upregulation in protein expression in S_Rs and S_RsPx samples (Fig. 5D) establishing the upregulation of PLA activity after Rs treatment that was observed in gene expression results.

New evidence has shown that phospholipids directly interact with NB-LRRs and mediate their localization to the PM, in order to recognize pathogen-derived effector proteins and induce immune responses [34]. This connection between phospholipids and LRRs made us hypothesize that phospholipid upregulation after Rs treatment in the S genotype could also be positively correlated with changes in the expression of these cell surface-localized receptors. Indeed, examination of our gene expression data of the Rs versus water comparison of the S genotype revealed that three leucine-rich receptors-like kinases, one G-type lectin S-receptor-like serine/threonine protein kinase and one F-box LRR were highly upregulated after Rs application (Supplementary Table S7). Since LRRs transduce information from the outer cell membrane to the nucleus of plant cells to eventually activate processes such as stress responses and plant resistance to diseases, this gene expression upregulation, in correlation with LRR interaction with phospholipids, further demonstrated the potential of Rs treatment in reinforcing signaling at the PM.

PA is a pivotal intermediate compound in glycerophospholipid biosynthesis and also a key signaling molecule regulating various processes, such as lipid metabolism, cytoskeleton dynamics, signal transduction, vesicular trafficking and hormone signaling, which are all tightly linked with defense signaling in plant-pathogen interactions [17, 32]. Except LPA conversion, PA can also be produced from the structural phospholipids Phosphatidylcholine (PC) or Phosphatidylethanolamine (PE) after hydrolysis by Phospholipase D (PLD) [18, 35]. PLD also has key roles in facilitating membrane traffic, meaning the mobility of soluble and membrane-related proteins with vesicular carriers from one membrane compartment to another [36]. We found accumulation of PE and PC (Fig. 5E and F) and PLD protein upregulation in S_Rs and S_RsPx samples (Fig. 5D), which come in agreement with the enriched gene functions of transmembrane activity and fatty acid metabolism, further confirming the upregulation of phospholipid pathway by Rs. Additionally, both PATATIN and PLD proteins were upregulated in S_Px sample, validating the induction of the phospholipid pathway after pathogen inoculation in the S genotype. Subsequently, to verify that the upregulated phospholipid pathway increased PA production, PA levels were quantified. Indeed, both pathogen inoculation and RS application caused significant upregulation of PA levels in all treatments of the S genotype, when compared to water control (S_W) samples (Fig. 5G). Notably, statistically significant increase of PA levels was found in the control IR plants (IR_W) and all other IR treatments. These findings supported the link between pathogen infection, inducer application and presence of inherent resistance.

### Pm-0 derived inherent phospholipid upregulation in the IR plants

**Figure 6:**
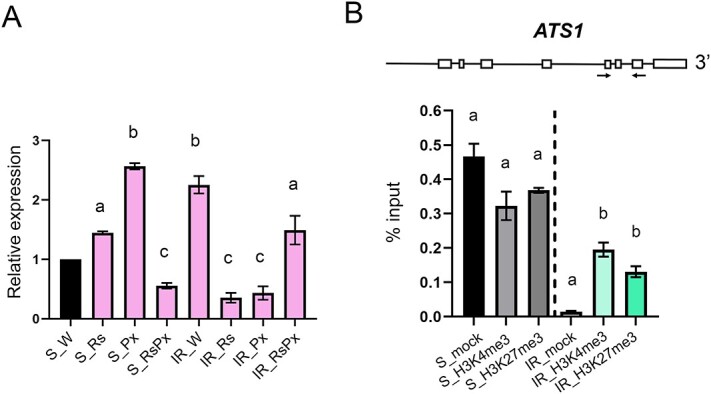
Epigenetic upregulation of Pm-0 G3P acyltransferase in IR genotype. (A) Relative expression analysis of *ATS1* was performed in leaves of the S and IR genotypes after Regalia® treatment, Px inoculation and combination of both treatments. Data represents the mean ± SEM of six biological replicates obtained from two independent experiments. *p*-values are determined performing one-way Analysis of Variance (ANOVA). Means were separated using the post hoc Dunnett’s multiple comparison test. Different letters indicate statistically significant differences at *p*-value ≤0.05. (B) ChIP on *ATS1* gene genomic region. The enrichment of H3K4me3 and H3K27me3 marks in the tested locus relative to percent input was determined by ChIP-qPCR in leaves of the S and IR genotypes at control conditions. Comparison of immunoprecipitated samples was performed to their mock controls. m: no antibody ChIP samples; H3K4me3: a-H3K4me3 ChIP samples; H3K27: a-H3K27me3 ChIP.

In the IR genotype, we did not observe any significant lipid metabolite accumulation after Rs treatment or Px inoculation (Fig. 5C-E). The IR_W samples displayed considerable PATATIN and PLD protein overexpression compared to all other tested samples (Fig. 5F). This intriguing result made us speculate that underlying IR specific genetic traits might regulate this enrichment since we did not detect any lipid-related upregulation in our transcriptomic findings. Analysis of IR’s genetic background in previous work [20] identified epigenetic upregulation of genes in *Pm-0* locus insertion, a genomic integrated region conferring resistance to Cucurbitaceae crops against *P. xanthii* [26]. Inside *Pm-0* locus, we identified *ATS1* gene that encodes for a G3P acyltransferase (GPAT) catalyzing LPA production [37–40]. To examine if *ATS1* is differentially expressed between genotype and treatments, qRT-PCR analysis was performed. Indeed, *ATS1* transcriptional examination revealed significant upregulation in IR_W compared to S_W genotype, while Rs treatment significantly downregulated its expression in the IR genotype (Fig. 6A). Moreover, pathogen inoculation caused significant upregulation of *ATS1* expression in S genotype substantiating the significance of this *Pm-0* locus gene in induction of the defense phospholipid cascade (Fig. 6A). We performed chromatin immunoprecipitation (ChIP) assay to search for epigenetic alterations and we found enrichment of H3K4me3 and H3K27me3 marks on the tested *ATS1* region only in IR_W genotypes (Fig. 6B).

## Discussion

Global food demand urge growers in agriculture to manage plant pathogens causing diseases with synthetic chemical pesticides. However, the widespread and extensive use of pesticides has adverse effects such as pollution of water and soil, pesticide residues in agricultural products, and development of pathogens’ resistance in a variety of crop/ pests combinations [41]. On this basis, sustainable agriculture is the only future solution. It is thus necessary to identify new methods and technologies for plant protection that are economically viable and effective in controlling diseases and pests to safeguard food security. Breeding for disease(s)-resistance in combination with new generation of plant protection products such as PDIs, is an environmentally friendly method of crop protection. Understanding the mode of action of such a heterogeneous group of plant protection products (with variant mode of action depending on the host-pathogen combination) is of pivotal significance to achieve acceptable, in practice, results. The use of PDIs will be increased in future crop protection strategies when knowledge on their potential effectiveness is broadened and their limitations are elucidated.

Powdery mildew is one of the most distractive diseases of cucurbits worldwide, and the causing agents *P. xanthii* [42], *Erysiphe cruciferarum*, and *Golovinomyces cichoracearum* [43] are obligate biotrophic fungi. Previous studies have shown that foliar applications of Rs extract in cucumber and courgette significantly reduced powdery mildew incidence and severity on leaves [21, 44–46], therefore increased fruit yield. The evidence show that Rs can prime resistance mechanisms including a network of diverse early signaling events such as rapid accumulation of ROS, ion flux changes, callose formation, changes in gene expression, production of stress-related hormones and resistance metabolites to enable plants combat pathogen infection [21]. We have found that the resistance mechanisms that were shown to be induced by Rs are highly similar to defense mechanisms that are activated inside plant cells after pathogen attack and infection. Indeed, our RNAseq results showed that in the S genotype after pathogen inoculation or Rs application, significant upregulation of serine/threonine kinases, lignin formation enzymes, transmembrane proteins, ion transporters, CoA synthases, and ligases were found, demonstrating that Rs activates plant defense mechanisms in a manner that mimics pathogen’s attack.

To activate the diverse components of the defense machinery, plants should have a signal transduction pathway that transmits signals coming from PRRs or NB-LRRs downstream, promoting signal perception to resistance activation. Nowadays, there is increasing evidence showing that lipids and lipid-related molecules, such as phospholipids, sphingolipids, fatty acids and jasmonates, play key roles in regulating defense-related signaling pathways [15, 47, 48]. Phospholipids are not exclusively structural components of biological membranes. Using phospholipid-based signaling, plant cells mediate the detection of extracellular signals. When an invading pathogen is detected, several phospholipid hydrolyzing enzymes are activated, which promotes the establishment of appropriate resistance responses [30]. Functional analysis of our transcriptomic results showed that Rs weekly application upregulated mainly the fatty acid metabolism, lipid biosynthesis and membrane transfer, demonstrating that extracellular signal perception through membrane lipid pathway is highly induced. This conclusion was confirmed with the metabolomic results where we detected substantial accumulation of the LPA, LPG, PC, PS, and PE metabolite classes in the S genotype leaves after Rs treatment with and without pathogen inoculation. PATATIN and PLD phospholipases were also upregulated after Rs treatment, reinforcing the enrichment of the lipid biosynthesis pathway. PATATIN, a protein with PLA activity, promotes PA turnover to LPA, while PLD produces PA from PC [49]. So, it is obvious that the PDI regulates PA dynamics and homeostasis in plant cells, from membrane component to signaling molecule. Additionally, we did not detect any lipid compounds in plants treated only with the pathogen, and this could be attributed to a moderate signaling response when compared to the lipid pathway upregulation after Rs application. Interestingly, pathogen inoculation on the Rs treated S plants led to reduction of LPA, LPG, and PC metabolite classes, suggesting that plant cells responded rapidly to pathogen attack by employing the accumulated metabolites. Similar reduction was also observed in the expressions of the corresponding lipid pathway genes in the RNAseq data. Our results are the first demonstration that induction of resistance by a botanical PDI, such as Rs, takes place through the phospholipid signaling in courgette. Moreover, we could speculate a broad function of this mechanism since we have identified induced phospholipid signaling pathway in Arabidopsis after the application of a chitosan-based inducer (unpublished data).

An intriguing result was the abundance of PATATIN and PLD proteins in the control IR leaves, even though lipid metabolite accumulation was not detected. As we already discussed, PATATIN, a PLA-like protein, and PLD regulate PA homeostasis and turnover in plant cells. Protein abundance in correlation with significant decrease in LPA and PC phospholipid classes indicate rapid synthesis and turnover of PA, a profoundly activated homeostasis waiting to respond to imminent pathogen infection. Nevertheless, the suppression of the phospholipid signaling pathway after Rs treatment or pathogen inoculation in the IR plants, is a subject that needs further examination.

Another major question that came to light was the trait of the IR genotype that enhances phospholipid pathway. Previous work has shown that IR courgette genotypes, including the one that was used in this study, bear genomic insertion of *Pm-0* locus, a genomic region that confers resistance to cucurbits against *P. xanthii* [20, 26] and that components of this region are epigenetically regulated in order to have activated the resistance mechanisms [20]. Detailed examination of the genes included in *Pm-0* locus revealed that *ATS1* gene has GPAT activity, catalyzing the acylation at sn-1 position of G3P to produce LPA. *ATS1* expression was upregulated in the S genotype after Rs treatment and pathogen inoculation and was also increased in the IR control plants. The ChIP assay showed enriched both H3K4me3 and H3K27me3 epigenetic marks on *ATS1* genomic region of the IR genotype, indicating a distinctive mode of *ATS1* expression regulation. The presence of both epigenetic marks could suggest a tight modulation of *ATS1* expression to balance the production of LPA and subsequent phospholipid signaling in IR plants.

There is always an open discussion on the possibility to breed for improved induced defense response. Responsiveness to PDIs depends on various factors and diversifies according to the plant genotype [50, 51]. Experiments on a combination of acibenzolar-S-methyl (ASM), β-aminobutyric acid and Cis-jasmonate PDIs in barley cultivars in controlled and greenhouse conditions showed great differences among genotypes in induced defense against *Rhynchosporium secalis* and *Blumeria graminis* f.sp. *hordei* [52]. Another study on tomato accessions and BABA showed that defense induction varied significantly among genotypes but was also dependent on the *Phytophthora infestans* isolate [53], adding more factors to the already complex mechanisms of induced defense. Here, we detected substantial differentiation in the molecular responses between the S and the IR courgette genotypes after Rs application. The RNAseq showed that IR plants do not respond to Rs treatment with or without pathogen inoculation, and the same results were observed in the metabolomics approach where no significant accumulation of lipid metabolite classes were detected. On the other hand, pathogen inoculation caused immediate upregulation of defense mechanisms, shown by the GO enrichment, a case that was not observed in the S genotype. The development of PDIs is becoming an emerging field of plant defense engineering technology. This popular new field enables scientists to control pests and diseases using molecular tools based on plant defense and could support new pesticides generation that is expected to become a new strategic industry with extensive developmental prospects [54]. On the other hand, breeding for a targeted defense response pathway could be an efficient alternative. There is an example of *A. thaliana* overexpressing the *NIM1* defense gene, encoding the NPR1 signaling protein, being more responsive to application of ASM [55].

The work presented here is the first report that Rs, a botanical PDI, induces phospholipid signaling in a genotype-related manner to subsequently activate plant defenses leading to new knowledge on the potential combination, in practice, of resistant hosts with compounds acting as plant defense inducers.

## Materials and methods

### Plant materials

In this work, the IR F1 hybrid Cordelia (Syngenta) and the S cultivar Kompokolokitho (Kompo, Hellenic Agricultural Organization-Demeter) were used. All experiments were performed in a greenhouse with controlled conditions (light/dark: 16 h/8 h, relative humidity 65%, 22°C). Plants were assigned to a completely randomized split-plot design, where each replicate consisted of six blocks with genotype as the main plot (24 plants) and foliar spray treatment as the subplot (12 plants).

### Pathogen inoculation

Inoculations with *Podosphaera xanthii* conidia were performed on 3-week-old plants by applying a freshly collected suspension of 1.5 x 10^5^ conidia ml^−1^ of the fungus with a sprayer, 2 d after Rs treatment.

### RNA extraction, sequencing, and bioinformatic analyses

Total RNA was extracted in triplicates for each treatment by NucleoSpin RNA Plant and Fungi (Macherey-Nagel). cDNA libraries were prepared using the TruSeq RNA Sample Prep Kit v.2 (Illumina) according to manufacturer’s instructions and sequenced using the NovaSeq 6000 platform (Illumina) by an external collaborator. Illumina reads were cleaned and aligned to the reference *C.pepo* MU-CU-16 genome v.4.1 [56] (http://cucurbitgenomics.org/v2/) by STAR v.2.5.3.a [57]. The aligned reads were processed by the Python package HTSeq to obtain gene read counts. Statistical significance of differential expressed genes was performed by the R package DESeq2 v.1.32.0 [58]. Differentially expressed were considered the genes that showed a threshold of log2FoldChange >1 and < −1. GO term enrichment analysis of DEGs was performed by Cytoscape [22].

### RT-qPCR

All treatments were performed as above. For each treatment, three plants were used. Total RNA was extracted with NucleoZOL (Macherey- Nagel) from two pooled leaves from each plant. 1 μg of total RNA was used for cDNA synthesis using the PrimeScript RT reagent Kit with gDNA Eraser (Takara). A StepOnePlus real-time thermal cycler (Applied Biosystems) was used for RT-qPCR with KAPA SYBR FAST qPCR kit master mix (KAPA Biosystems). Each reaction was run in duplicate and repeated twice at: 10 s at 95°C, 40 cycles of 95°C for 5 s, 60°C for 30 s, and final 5 s at 95°C with 30 s at 60°C. The *EF1a* gene was used for normalization. The list of primers used in this work is presented in Supplementary Table S8.

### Western blotting

Total proteins were isolated from the same samples as for expression analysis, with an extraction buffer containing NP-40, SDS, and Triton X-100. 2 min total sonication steps were performed to the isolated proteins. Equal protein amounts were loaded and resolved on an 8% SDS–polyacrylamide gel and then transferred to Immobilon- P membrane (Millipore). Rabbit anti-PATATIN (Agrisera), rabbit anti-PLD (Agrisera) and rabbit anti-ACTIN (Agrisera) were the primary antibodies used for immunoblotting. Before incubation with the secondary HRP-conjugated goat anti-rabbit IgG (Carl Roth), the membranes were washed three times. The Clarity Western ECL Substrate (Bio-Rad) was used for the detection of the chemiluminescence signal, and ChemiDoc imaging system (Bio-Rad) was used for image acquisition.

### ChIP-qPCR assay

ChIP was performed as described previously [59]. Three leaves from plants of each genotype in control conditions were fixed and 1 g of tissue was used for chromatin isolation. Fragmented chromatin was immunoprecipitated either with anti-H3K4me3 or anti-H3K27me3 antibodies (Cell Signaling). For the ChIP–qPCR analysis, a fragment containing an *ATS1* genomic region was used. Quality examination of the immunoprecipitated DNA was performed by detecting the euchromatic *EFL1a* gene (Supplementary Fig. S2). The list of primers used in this work is presented in Supplementary Table S8.

### Metabolomics analysis

Leaves from three plants from all treatments were collected. Injections of leaf extracts were performed in a Q Exactive Orbitrap Mass Spectrometer (Thermo Scientific) following a previously described metabolomics protocol [60]. Analysis was performed employing a Dionex Ultimate 3000 UHPLC system (Thermo Scientific). A Hypersil Gold UPLC C18 (2.1 × 150 mm, 1.9 μm) reversed phased column (Thermo Scientific) was used for the analysis. Mobile phase A was aqueous with 0.1% (v/v) formic acid and mobile phase B was acetonitrile. The flow rate was 0.220 ml/min and the injection volume 5 μl. The column temperature was kept at 40°C while the temperature of the automatic sample tray was set at 4°C. Both negative and positive ionization modes were performed at HESI. Data acquisition was performed in Xcalibur 4. The raw MS data were converted to ABF format and were inserted to MS-DIAL for peak peaking, alignment, integration, and retention time correction according to optimized parameters. The resulting output tables of metabolites were subjected to multivariate statistical analysis. The data that exhibited variable importance in projection scoring were compared with Lipid Maps database in MS-DIAL, while applying 5 ppm m/z tolerance.

For PC metabolite class quantification, the PC Colorimetric Assay Kit (Cayman Chemical) was used, following manufacturer’s instructions.

For PA metabolite quantification, the Total Phosphatidic Acid Assay Kit (Cell Biolabs, Inc) was used, following manufacturer’s instructions.

## Supplementary Material

Web_Material_uhae190
