# Supplementary material for: Phospholipid production and signaling by a plant defense inducer against Podosphaera xanthii is genotype-dependent
Source: Hortic Res. 2024 Jul 12;11(9):uhae190. doi: 10.1093/hr/uhae190 (PMC11377184; doi:10.1093/hr/uhae190)
Supplement: Web_Material_uhae190 [file web_material_uhae190.zip › Supplementary Data Table S7.docx]

**Supplementary Data Table S7**: Significantly upregulated leucine-rich repeat receptors in the Rs versus water comparison of the S genotype.

| gene | description | Log2FC | padj |
| --- | --- | --- | --- |
| Cp4.1LG17g05270 | Leucine-rich repeat protein kinase family protein | 8.738333 | 2.58E-07 |
| Cp4.1LG09g00610 | G-type lectin S-receptor-like serine/threonine-protein kinase At5g24080 isoform | 3.485067 | 0.006626 |
| Cp4.1LG06g08830 | LRR receptor-like serine/threonine-protein kinase GSO1-like | 1.91913 | 0.01706 |
| Cp4.1LG17g08780 | F-box/LRR-repeat protein 20-like isoform X1 | 1.759621 | 0.013297 |
| Cp4.1LG03g14260 | putative leucine-rich repeat receptor-like protein kinase At2g19210 | 1.459812 | 0.026336 |
