# Supplementary material for: Phospholipid production and signaling by a plant defense inducer against Podosphaera xanthii is genotype-dependent
Source: Hortic Res. 2024 Jul 12;11(9):uhae190. doi: 10.1093/hr/uhae190 (PMC11377184; doi:10.1093/hr/uhae190)
Supplement: Web_Material_uhae190 [file web_material_uhae190.zip › Supplementary Figure Legends.docx]

**Supplementary Figure Legends**

**Supplementary Data Fig.1:** **Transcriptomic analyses of the samples used in this research**. **A,** Heatmap of the gene transcript abundance in the samples used in this research. condition: *R. sachalinensis* or water (control) treatment, pathogen: *Podosphaera xanthii* inoculation or water (control) treatment. Color scale bar indicates transcript abundance of each gene in each sample. **B,** Volcano plots show abundantly and significantly expressed transcripts in the tested differential expression analyses. Dots indicate points-of-interest that display fold-changes (x axis) and statistical significance (-log10 of *P* value, y axis) in transcripts. Pink dots indicate transcripts with fold-changes of one or more and *P* adjusted values of less than 0.05 and are transcripts with significant changes between the transcriptomes. Black dots represent transcripts with non-significant changes between the transcriptomes.

**Supplementary Data Fig.2**: **qPCR for the euchromatic EFL1a gene in ChIP input samples and final chromatin samples after immunoprecipitation in ChIP assay.** *EFL1a* DNA is highly detected in the isolated chromatin samples from each genotype (S and IR input samples), while no amplicon is detected in the chromatin samples after immunoprecipitation with no antibody (m_ChIP samples) or H3K4me3 (4_ChIP samples) and H3K27me3 antibodies (27_ChIP samples). The experiment was performed in all three biological replicates of the ChIP assay with similar results.
