# Supplementary material for: Phospholipid production and signaling by a plant defense inducer against Podosphaera xanthii is genotype-dependent
Source: Hortic Res. 2024 Jul 12;11(9):uhae190. doi: 10.1093/hr/uhae190 (PMC11377184; doi:10.1093/hr/uhae190)
Supplement: Web_Material_uhae190 [file web_material_uhae190.zip › Supplementary_Data_Table_S8.docx]

| Primer name | Sequence 5’-3’ |
| --- | --- |
| Inositol_phospho_F | ACTATTGGCAAAGTTCCAACAG |
| Inositol_phospho_R | AGACTTAATGCACATGATCCAC |
| Patatin_F | AATAATCCCACATTAGCAGCC |
| Patatin_R | AGTCCCCAGAGATAGAACAAG |
| qATS1_F | AGGAGGTTAGGGAGGCATAC |
| qATS1_R | GACAAAGAGACATCCGCAG |
| ATS1_chip_F | ATGACATCATGCCACCTCC |
| ATS1_chip_R | ACACAGAATCAAACAGAGCC |
| EF1a_F | ACGCTCTCCTTGCTTTCAC |
| EF1a_R | CGACACTTCCTTCACGATTTC |
| Phospholipase_A1_F | ACGAGATGGGTGTGAAGAC |
| Phospholipase_A1_R | GTACAGCCAATTCAGCTTCC |
| Cinnamoyl_CoA_F | TCCATTCCAGAAATTCAACTCC |
| Cinnamoyl_CoA_R | CGAAGCAAATGTATCTGCCG |

**Supplementary Table 7**. List of primers used in this study.
