# Supplementary figures and images for: Phospholipid production and signaling by a plant defense inducer against Podosphaera xanthii is genotype-dependent
Source: Hortic Res. 2024 Jul 12;11(9):uhae190. doi: 10.1093/hr/uhae190 (PMC11377184; doi:10.1093/hr/uhae190)

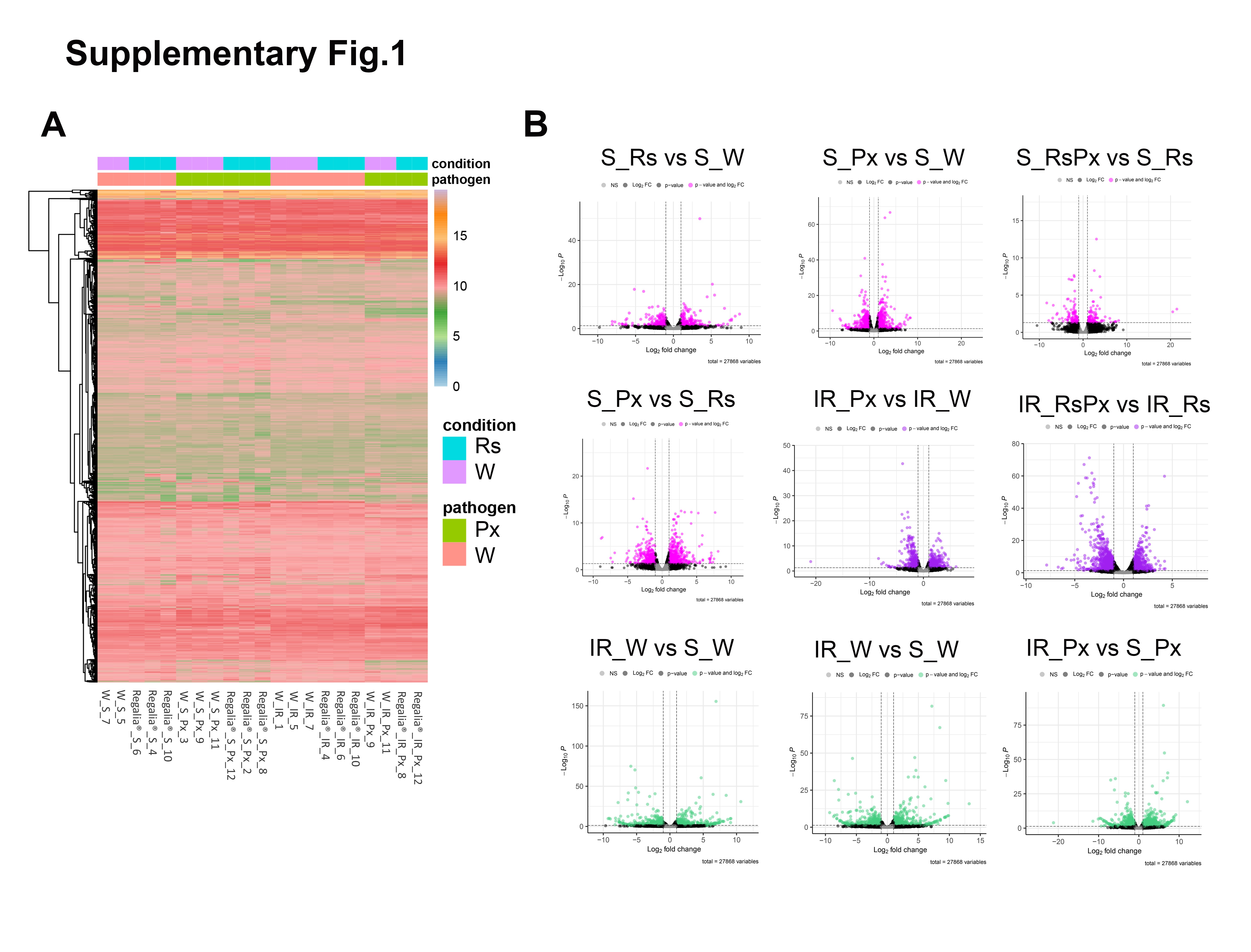

Supplement: Web_Material_uhae190 [file web_material_uhae190.zip › sup_fig_1.jpg]

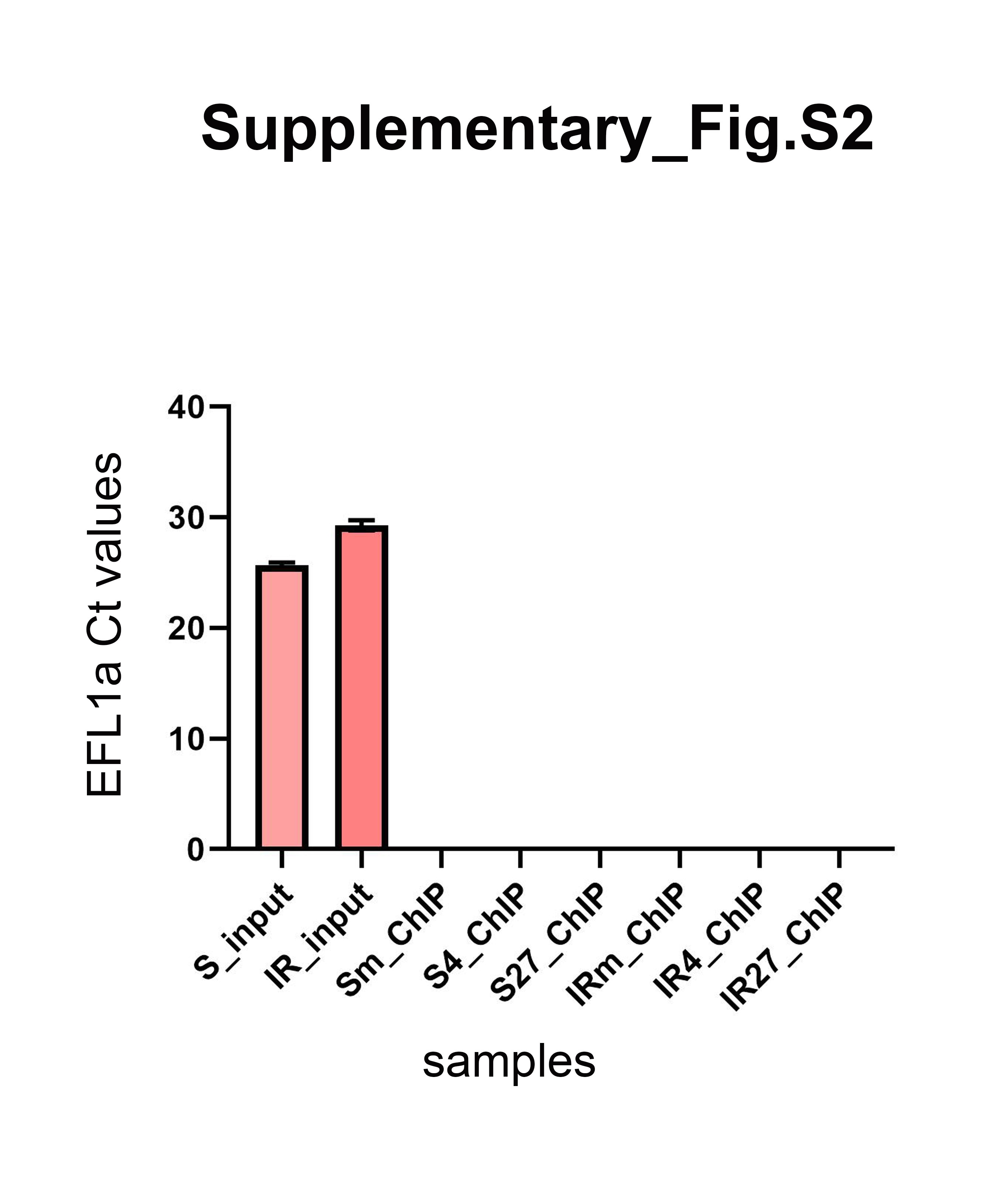

Supplement: Web_Material_uhae190 [file web_material_uhae190.zip › sup_fig_2.jpg]
